# Evidence supporting the conceptual framework of cancer chemoprevention in canines

**DOI:** 10.1038/srep26500

**Published:** 2016-05-24

**Authors:** Tamara P. Kondratyuk, Julie Ann Luiz Adrian, Brian Wright, Eun-Jung Park, Richard B. van Breemen, Kenneth R. Morris, John M. Pezzuto

**Affiliations:** 1 Department of Pharmaceutical Sciences, The Daniel K. Inouye College of Pharmacy, University of Hawaii at Hilo, 34 Rainbow Drive, Hilo, HI 96720, USA; 2 Department of Pharmacy Practice, The Daniel K. Inouye College of Pharmacy, University of Hawaii at Hilo, 34 Rainbow Drive, Hilo, HI 96720, USA; 3 Department of Medicinal Chemistry and Pharmacognosy, College of Pharmacy, University of Illinois, 833 S. Wood Street, Chicago, IL 60612, USA

## Abstract

As with human beings, dogs suffer from the consequences of cancer. We investigated the potential of a formulation comprised of resveratrol, ellagic acid, genistein, curcumin and quercetin to modulate biomarkers indicative of disease prevention. Dog biscuits were evaluated for palatability and ability to deliver the chemopreventive agents. The extent of endogenous DNA damage in peripheral blood lymphocytes from dogs given the dietary supplement or placebo showed no change. However, H_2_O_2_-inducible DNA damage was significantly decreased after consumption of the supplement. The expression of 11 of 84 genes related to oxidative stress was altered. Hematological parameters remained in the reference range. The concept of chemoprevention for the explicit benefit of the canine is compelling since dogs are an important part of our culture. Our results establish a proof-of-principle and provide a framework for improving the health and well-being of “man’s best friend”.

Since the average lifespan of a healthy canine is shorter than the average lifespan of a healthy human being, people commonly experience the loss of their companion animals^1^. More than half of dogs over 10 years of age are likely to develop cancer^2^. Although treatment measures are available for canine neoplasia, these procedures are typically expensive and results are variable based on the overall stage of the disease. As an example, once diagnosed with osteosarcoma, the median survival time without treatment, with amputation alone, or with palliative radiotherapy alone, is 4 months. With surgery and chemotherapy this is increased to only 10 months^3^. In addition, while human therapies are often highly subsidized, the cost of treating service animals and pets generally falls wholly on the owners. The expense associated with treatment options varies, but may range from $6,000–10,000 for chemotherapy, $5,000–7,000 for radiation, and $2,500–6,000 for surgery^4^. Medical expenses of this magnitude are likely to pose undue hardship on millions of people nationwide.

On the other hand, chemoprevention is an attempt to use nontoxic natural and synthetic substances or their mixtures to intervene during the relatively early stages of carcinogenesis, before invasive characteristics are manifested^5^. Since carcinogenesis is regarded as a multistep process (e.g., initiation, promotion, progression)^6^, in principle, blocking or inhibiting any of these stages could help to prevent or delay tumorigenesis. Further, consistent with epidemiological studies suggesting that a reduced risk of cancer is associated with consumption of vegetables and fruits^7^,^8^,^9^,^10^,^11^, many agents with cancer chemopreventive potential are naturally occurring phytochemicals^12^,^13^,^14^. The etiologic basis of cancer, as well as most age-related diseases, is complex, but it is generally agreed that oxidative stress plays a role^15^. Damage to DNA by oxidative stress is well known^16^. The aim of this study was to assess whether dietary intake of some common dietary chemopreventive agents could affect DNA damage and the expression of genes related to oxidative stress with dog lymphocytes.

In enrolling canine pets as the subjects of the study, our objective was to perform a relatively short-term and non-invasive trial. Accordingly, lymphocyte DNA was used as a surrogate target to evaluate oxidative stress *ex vivo*. As described in the literature, this procedure has been used for assessing both the carcinogenic and anti-carcinogenic potential^17^ of test substances and, although lymphocytes are used for the observation, it may be surmised that other cell-types susceptible to carcinogenic insult could mount a similar response^17^. Since the *ex vivo* protective response of the lymphocyte is evaluated, and the chemopreventive agent is administered orally, it is logical to assume any positive response elicited by the agent would be through an indirect mechanism such as induction of cytoprotective enzymes. Thus, we determined expression profiles with an array designed for canine oxidative stress genes.

In order to conduct the canine trial, we formulated and manufactured a biscuit containing five known natural product chemopreventive agents: resveratrol, ellagic acid, curcumin, genistein and quercetin. When administered as single agents, some studies with dogs have been reported in the literature. For example, plasma levels of genistein were determined in Beagle dogs treated with immediate and extended release tablets^18^, dogs were treated with curcumin for the potential therapeutic management of osteoarthritis^19^, and quercetin, in combination with clopidogrel, affected the activity of P-glycoprotein^20^. Of course, many preclinical studies have been performed with dogs to assess potential toxicity as a prelude to conducting human trials, such as with resveratrol^21^,^22^,^23^.

The combination of chemopreventive agents in our formulation is unique and therefore has not been tested before. As described herein, a number of factors were taken into account in making the selections, but notably, each agent has been evaluated singly in human clinical trials^24^,^25^,^26^. This gave us confidence in conducting a canine trial. Further, rather than selecting a single agent, we were enthusiastic about testing a group of active compounds since it is anticipated that combination cancer chemoprevention will produce a synergistic response^27^.

## Results

For the conduct of this work, a dog biscuit formulation was devised that contained a combination of resveratrol, genistein, ellagic acid, curcumin and quercetin as active chemopreventive agents. The study design involved two phases. The first phase was to evaluate the palatability and acceptability of the placebo treats. Over a period of three weeks, eight healthy dogs were given the placebo treat twice per day. This assessment was simply to determine whether the dog liked/disliked the taste, texture, etc., of the biscuit, the owner’s ease of administration, and the dog’s motivation to eat the biscuit. The owners observed the behaviour of the dog to insure the biscuits were well tolerated with and without active ingredients to ensure their delivery. The dogs consumed the biscuits following the prescribed schedule. Some owners used masking techniques.

Following a two-month resting period, the second phase of the study was launched with the same canine subjects. Over a period of three weeks, the diets of the dogs were supplemented with two biscuit treats containing the chemopreventive agents (Table 1), one biscuit by mouth twice daily approximately 12 hours apart each day. Dogs did not experience any overt toxicity while enrolled in trial. There were no reported changes in bowel habits (diarrhea or constipation), no abdominal bloating, and no gastrointestinal bleeding. However, some minor gastrointestinal effects were reported by the owners. Of the eight canine subjects enrolled in the study, one owner reported decreased appetite while on the biscuit, one owner reported mustard-colored stools while on the biscuit, and three owners reported vomiting following administration of the biscuit that was sporadic.

Haematological parameters were assessed and all values were observed to fall within the normal range attesting to a general state of good health both before and after dietary supplementation. Comprehensive blood profiles did not show any difference between the first and second phases of the study falling outside of the normal range (Table 2). However, there was a significant increase in the level of serum phosphorous. The reason for this increase is not known. However, a low magnitude change in a single marker wherein the total level still remains in the reference range is not likely to be of clinical significance. In sum, these data suggest the treatment protocol did not lead to any type of overt toxicity.

The same was true when comparing baseline and post-treatment blood counts (Table 3). It is notable, however, when comparing these two groups, there was a statistically significant increase in the number of monocytes after the dogs were given the dietary supplement for the treatment period. Although remaining in the reference range, the monocyte number was found to be significantly higher in the second phase of the study (Table 3). This empirical observation is worthy of further investigation.

Serum was isolated from whole blood obtained during the two phases of the trial and analysed by LC-MS/MS. The chemopreventive agents used for this are not normally added to dog chow, and selected dosages were well within safety margins based on previous work conducted mainly in human clinical trials. At the end of this first (placebo) phase of the trial, no resveratrol, resveratrol metabolites, curcumin, curcumin metabolites, or ellagic acid were detected in serum. Genistein-4′-glucuronide (327.7 ± 211.13 ng/mL) and genistein sulfate (961 ± 680.1 ng/mL) were detected in serum of six dogs. It is likely the genistein metabolites were derived from the dog chow, which can contain soybean products. Quercetin levels were below detectable levels in five dogs, but three dogs had quercetin concentrations of 2 or 3 ng/mL. Quercetin occurs in many botanicals and was also probably a constituent of the dog chow (Table 4). After a two-month rest (wash-out) period, the dogs were started on the second phase of the trial by administering two biscuits per day containing the chemopreventive agents. The second phase lasted 3 weeks. Resveratrol and its metabolites were detected in serum of all dogs but one during the second phase. The most abundant form of resveratrol in dog serum was resveratrol-3-sulfate. Much higher concentrations of genistein metabolites were observed during the second phase (genistein-4′-glucuronide, 7083.74 ± 128.18 ng/ml; genistein-7-glucuronide, 861.74 ± 329.61 ng/ml), with the sulfate being dominant (genistein sulfate, 26958.61 ± 2300 ng/ml) (Table 4). A diet rich in genistein has been associated with lower rates of prostate and breast cancer^28^. When administered orally, genistein is rapidly absorbed and metabolized in humans and animals as are other isoflavones^29^. In the current study, no unconjugated genistein was detected after either the first or the second phases (Table 4). No ellagic acid or curcumin was detected in serum, although curcumin glucuronide was detected at low levels in serum of two dogs after the second phase.

**Figure 1:**
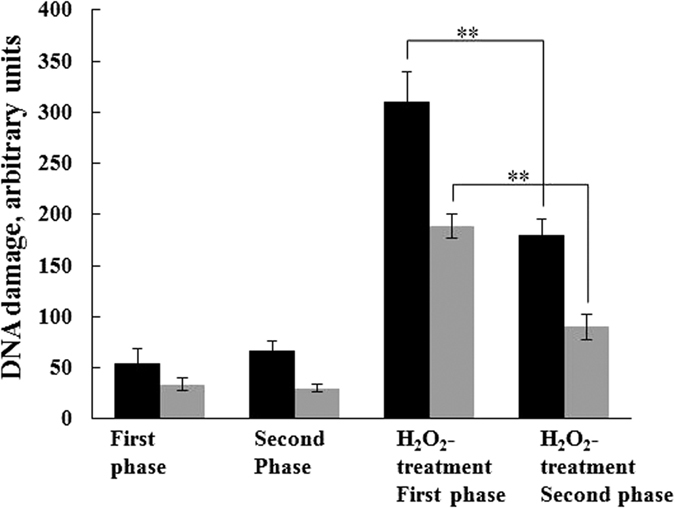
Chemopreventive supplements inhibit H_2_O_2_-induced DNA strand breakage in dog lymphocytes. Lymphocytes were washed, suspended in 1% (w/v) LMP agarose and pipetted onto frosted glass microscope slides. For H_2_O_2_ treatment, 10 μl of 1 mM H_2_O_2_ were added to 90 μl of cells in PBS (100 μM final concentration); after 5 min on ice, the cells were collected by centrifugation and applied to slides. Slides were washed three-times with buffer (40 mM HEPES-KOH, 0.1 M KCl, 0.5 mM EDTA, 0.2 mg/ml BSA, pH 8) and incubated for 45 min at 37 °C with either 50 μl buffer or endonuclease III in buffer (1 μg protein/ml). Lymphocytes were stained with SYBR Gold and image analysis was performed using a Leica confocal microscope (Fig. 2). Gray bars are without endonuclease treatment, black bars are with treatment. **p < 0.01, *t*-test (n = 16).

Starting with whole blood from the canine subjects, peripheral blood lymphocytes were isolated. Two measures of DNA damage were evaluated: endogenous DNA damage (DNA damage without H_2_O_2_ exposure) and H_2_O_2_-inducible DNA damage. We did not find any statistically significant difference in endogenous lymphocyte DNA damage between the two phases of the study (Fig. 1). In dog lymphocytes obtained during the first phase of the trial, H_2_O_2_-induced DNA strand breakage increased from 54 ± 15 to 310 ± 30 units. Remarkably, in second phase of the trial, following administration of the dietary supplement, lymphocyte DNA damage induced by treatment with 100 μM H_2_O_2_ was significantly decreased from 310 ± 30 to 180 ± 16 units (Fig. 1, grey bars). For further confirmation of these data, cell preparations were treated with endonuclease III and the measurements were repeated. Levels of endogenous oxidized pyrimidines did not differ during the first or second phases of the trial. However, H_2_O_2_ treatment increased the level of oxidized pyrimidines, and consumption of the dietary supplement protected lymphocytes against pyrimidine oxidation (Fig. 1, black bars).

**Figure 2:**
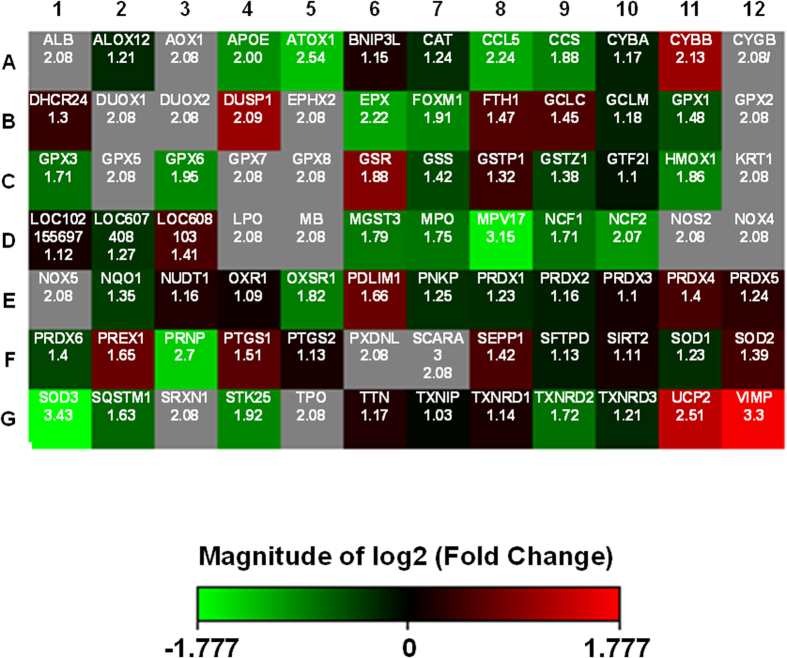
Relative expression levels of oxidative stress-related genes in dog lymphocytes. Lymphocytes were isolated from the same dogs after the two phases of the study. Total RNA was extracted and reverse-transcribed to cDNA. The resultant cDNA template was mixed with RT2 SYBR Green qPCR Mastermix and distributed equally as 25 µl aliquots in each well of the customized array plate. After the initial incubation at 95°C for 10 min to activate HotStart Taq DNA polymerase, real-time quantitative PCR was performed with the thermal cycling conditions as 40 cycles at 95°C for 15 sec followed by 60°C for 1 min. The fluorescence signal from SYBR Green intercalating into double-stranded DNA was recorded at the end of each elongation phase. The fold changes of 84 genes between the two phases of the study were analyzed using the web-based software provided by Qiagen (Data Analysis Center). The heat map represents fold changes in gene expression between control (the first phase) and dietary intervention (the second phase) groups. Red, green, or black squares in the heat map denote up-regulated, down-regulated, or unchanged, respectively, compared to the control group.

To further explore the ramifications of chemopreventive agent administration, total RNA was extracted from lymphocytes, cDNA was generated, and evaluated using a custom made PCR array relevant to dog oxidative stress (Table 5). Of the 84 genes evaluated, the expression of 11 was found to be significantly altered following administration of the dietary supplement (Table 6). The expression of five genes, including *CYBB, DUSP, GSR, UCP2* and *VIMP*, were significantly increased, whereas those of six genes, including *ATOX1, CCL5, EPX, MPV17, PRNP* and *SOD3*, were down-regulated (Fig. 2). Among these, *VIMP* and *MPV17* were most highly up- and down-regulated, respectively.

## Discussion

In situations wherein a tumor is diagnosed and completely excised prior to invasion or metastatic spread, treatment should be definitive. In canine cases, this may involve amputation or radical surgery. However, the situation is even worse when presented with malignant metastatic disease wherein life expectancy is reduced and treatment options are much more limited. Consequently, avoiding all such situations, ostensibly by means of cancer chemoprevention, remains a compelling approach. Despite issues associated with the development of cancer chemopreventive agents, proof-of-principal has been established with drugs such as tamoxifen and finasteride^30^. In nearly every case, for the discovery or development of new cancer chemopreventive agents, laboratory animals (primarily rodents) have been used as models. In more advanced stages of development, additional animals, including canines, are used to establish pharmacokinetics (absorption, distribution, metabolism, and excretion), dosage forms, toxicity, etc. Nonetheless, the ostensible goal of such work is to provide agents for the prevention of cancer with human beings.

Similar to humans, dogs manifest a broad range of cancers such as melanoma, non-Hodgkin lymphoma, osteosarcoma, soft tissue sarcoma, and prostate, mammary, lung and colorectal carcinomas. Approximately 1 in 3 dogs will be diagnosed with cancer during their lifetime, and cancer currently accounts for about half of the deaths of all dogs older than 10 years^2^,^31^. Of course from a humanitarian point-of-view prevention of canine cancer is gratifying, but owners of pets and service animals would also benefit. Thus, in this study, rather than using the canine as a model for human beings, our goal was to focus on potential benefits for the animal.

This is the first report to document oral administration of this schedule of combined chemopreventive agents in a canine study. The chemopreventive formulation devised for this study is somewhat arbitrary but based on a variety of pragmatic and theoretical considerations. First of all, it was of interest to select agents that (1) are natural products, (2) ostensibly function by pleotropic mechanisms (e.g., inhibit various stages of carcinogenesis such as initiation, promotion, and progression, either singly or in combination), (3) have well-known safety profiles or generally recognized as safe (GRAS) status, (4) have been extensively reported in the scientific literature as chemopreventive agents, and (5) are undergoing or have undergone evaluation in human clinical trials. The following agents were judged as having met these criteria: Reveratrol (‘typical’ representative dose, 1 g/d/person), lycopene (15 mg/d), α-tocopherol (50 mg/d), l-selenomethionine (0.2 mg/d), ellagic acid (250 mg/d), indole-3-carbinol (400 mg/d), sulforaphane (50 mg/d), quecetin (1 g/d), allicin (3 mg/d), genistein (500 mg/d), daidzein (300 mg/d), curcumin (1 g/d), and EGCG (300 mg/d). At this point in the selection process, some agents were eliminated since adequate quantities may be found in the average diet (e.g., vitamin D, selenium, and α-tocopherol), high expense or issues with general availability (e.g., sulforaphane), poor organoleptic properties (e.g., allicin), or innate difficulty in the biscuit production process.

In the end, a combination including resveratrol, genistein, ellagic acid, curcumin and quercetin was selected for administration at the dosage levels summarized in Table 1. The rationale of the dosage selection is described in Methods (Dog biscuit production). Admittedly, additional or alternative agents could have been included in this trial. Nonetheless, in addition to the rationalization presented above, another advantage was that each of the test agents is known to function by pleiotropic mechanisms of action that are relevant to disease prevention. For example, resveratrol has been reported to exert antioxidant, anti-inflammatory, anti-infective, cardioprotective, neuroprotective, antiobesity, and chemopreventive activities^32^,^33^,^34^,^35^. It has not been evaluated for cancer chemoprevention in dogs, but analytical work for the determination of *trans*-resveratrol and derivatives in dog plasma has been described^21^,^22^, as well as preclinical toxicity studies^23^. Ellagic acid occurs in many foods and has antioxidant, antibacterial, antiviral and cancer-preventing properties^36^. It exerts anticancer effects in various cancer cell lines and animal tumor models^37^. Curcumin, a natural polyphenolic antioxidant can mediate a large number of biological responses^38^. Some studies have reported pharmacokinetics in dogs, and stabilizing curcumin with phosphoric acid allows accurate quantitative determinations of curcuminoids in dog plasma^39^. The phytoestrogen genistein is found in soy-based products. It has been suggested that genistein can prevent both prostate and breast cancer^28^, particularly in Southeast Asia populations where soy products are consumed at high levels^40^. A LC-MS-MS method has been developed for the determination of genistein in dog plasma following oral administration^18^. Finally, quercetin mediates diverse anti-cancer effects^41^. It is a pleiotropic molecule with limited toxicity on normal cells. Simultaneous targeting of multiple pathways may help to eliminate malignant cells and retard the onset of drug resistance^42^.

The development and production of a biscuit-type dosage form for relatively large canines presented some challenges since it was important to preserve the integrity of the chemopreventive agents. Thus, the common practice of baking the final product was avoided. During the development phase, many formulations were designed to form durable biscuit-type tablets and changes were made in an effort to reduce bellyband cracks and improve compressibility, compatibility, and flavour. The compaction of each active ingredient was individually tested, which revealed that the compounds themselves aid the compression of the tablets and helped balance the unfavourable compaction characteristics of the other biscuit ingredients.

Another issue that will ultimately be faced in developing a canine chemopreventive product is expense. Based on the quantities and price schedule for the chemopreventive agents purchased and used in this study, the expenditure was $1.65/biscuit. It is difficult to know an amount consumers would find to be acceptable for such as product, but given the high expense associated with treatment (*vide supra*) and the gratification accompanying disease prevention, an expenditure of this magnitude seems reasonable. Another aspect related to expense is when the dog would begin biscuit consumption. While it is not possible to provide an answer based on existing evidence, since it has been noted that more than half of dogs over 10 years of age are likely to develop cancer^43^, it would seem that early administration would be beneficial. One suggestion would be to start consumption at the time of full growth, which would be roughly 1 year for smaller breeds, and 2 years for larger breeds.

Using the formulation devised for this study, we were able to demonstrate detectable serum levels of resveratrol, genistein metabolites, and quercetin (and metabolites), indicating they cross canine enterocytes and enter blood circulation. This promoted a protective effect on oxidative DNA damage. Since lymphocytes are readily assessable and play important roles in both the physiological and pathological processes, it is reasonable to regard them as a useful indicator for oxidative DNA damage^44^, in humans^45^ as well as canines^46^ and other species^17^. Our general hypothesis is that lymphocytes serve as a surrogate for other cell types, and protection from DNA damage is consistent with cancer chemoprevention.

The mechanism of protection remains to be defined, but to provide some indication of the effect of the chemopreventive formulation on oxidative stress gene expression, array analysis was performed. Of the up-regulated genes, three belong to oxidative stress responsive group (DUSP1, GSR, VIMP), CYBB is an antioxidant, and UCP2 is related to superoxide metabolism. Many selenoproteins (VIMP) participate in intracellular redox homeostasis and play antioxidant roles^47^. The expression of selenoprotein S (SelS) is related to inflammation and insulin resistance suggesting that SelS may provide a link between inflammation and oxidative stress pathways through its role as an antioxidant^48^. Down-regulated genes are represented by two genes (ATOX1 and MPV17) associated with ROS metabolism, two oxidative stress responsive genes (CCL5 and PRNP), and two others from the antioxidant group (SOD3 and EPX)^31^,^49^,^50^,^51^,^52^ (Table 6). Overall, in the context of gene expression, up-regulation (e.g., GSR and UCP2) and down-regulation (e.g., SOD3 and the transcription factor for SOD3, Atox1)^53^ may improve antioxidant response or decrease inflammation.

In order to assess the general health of the animals, comprehensive blood profiles were examined during the first and second phases of the study. No changes of veterinary concern were observed, but the population of monocytes and the concentration of phosphorous were found to be increased after the dogs were given the dietary supplement for the treatment period. Nonetheless, every parameter determined remained in the reference range.

In sum, the results reported herein show significant promise and support the notion of perfecting a nontoxic dietary supplement that can be of value in enhancing the health and well-being of “man’s best friend” the canine. Of course this would be a meaningful accomplishment for the dog him- or herself, but owners of domestic pets and service animals would also derive great benefit. Further, it is encouraging that regulatory requirements would be less stringent than those mandated for human consumption, and ultimately, the work with canines may provide some insight relevant to human disease prevention.

## Methods

### Chemicals and reagents

The sources of the chemopreventive agents used in this study are listed in Table 1. Prior to conducting the work, all agents were assessed for purity using LC-UV-MS with a Shimadzu (Kyoto, Japan) IT-ToF high resolution hybrid mass spectrometer equipped with reversed phase HPLC, electrospray and an in-line UV absorbance array detector (Table 1). HPLC-grade methanol and acetonitrile were purchased from Sigma-Aldrich (St. Louis, MO) or Thermo-Fisher (Waltham, MA). Distilled water, prepared by a Milli-Q water purification system from Millipore (Milsheim, France) was used throughout the study. Endonuclease III from *E.coli*, recombinant, Histopaque-1077, Hank’s balanced salt solution (HBSS), dimethylsulfoxide (DMSO), phosphate buffered saline (PBS), and hydrogen peroxide (H_2_O_2_) were purchased from Sigma-Aldrich. Trizol reagent was purchased from Invitrogen (Life Technologies, Foster City, CA). SYBR Gold nucleic acid stain was from Life Technologies (Carlsbad, CA), Trypan blue solution, 0.4% was purchased from Gibco (Grand Island, New York). Comet assay kit for single cell gel electrophoresis was purchased from Trevigen (Gaithersburg, MD). RT^2^ Profiler custom made PCR Array: CAPF12480 related to dog oxidative stress was purchased from QIAGEN-Frederick (SABiosciences, Valencia, CA) (Table 5). All other chemicals were of analytical grade.

### Canine subjects

Prior to the initiation of dietary experiments, study protocols were reviewed and approved by the University of Hawaii Institutional Animal Care and Use Committee (IACUC). The methods were carried out in accordance with the approved guidelines. At each stage of the study, the dogs were examined by the same veterinarian (JALA).

Dogs were client-owned and participated at the owner’s consent. Each dog remained on their individual, normal diet for the duration of the study; no special diet was introduced except for that of the biscuit. Inclusion criteria were as follows: Greater than 1 year of age; in the weight range of 50–100 pounds. In addition, the subjects were deemed healthy via physical examination by the veterinarian. Any dog not meeting the inclusion criteria was excluded from the study. The eight study participants ranged in age from 2–12 y (median: 6 y), and in weight from 49.4–72.4 lbs (median: 60.4 lbs; 27.45 kg). The participants were of the following breeds: Golden retriever, Border collie mix, New Finland/English setter mix, Golden retriever/Labrador mix, Labrador/Australian shepherd mix, German shepherd/Labrador mix, Labrador, and Labrador mix.

### Dog biscuit production

Since we were not in a position to custom manufacture biscuits of various sizes the following assumptions were made in the design phase of the study. First of all, in the more typical situation of converting an animal dose (mg/kg) to a human-equivalent dose (mg/kg), one approach is to divide the animal dose by a factor of 1.8^54^. Here, being presented with doses taken by human beings, our goal was to determine a canine dose by extrapolation. For example, a value of 1 g/day/person was selected for resveratrol, or around 16 mg/kg body weight. Applying the conversion factor of 1.8, the canine dose becomes 29 mg/kg, and assuming a body weight of 20–30 kg, the daily dosage would be 580–870 mg. These are very rudimentary conversions, and the safety profiles of the test agents are extremely broad, but weighing on the side of being conservative, a daily dose of 500 mg was selected. A similar approach was used for selecting the dosages for the remaining chemopreventive agents used in this study.

For the formulation of dog biscuits, the base mix contained wheat flour (0.62 kg), corn meal (0.17 kg), eight beef bouillon cubes (4.136 g each), and water (225 ml). Dry ingredients were weighed out and added to a planetary mixer and mixed for three minutes. Water was added dropwise over 75 seconds. The mixture was then baked for 60 min at 60 °C yielding a total batch weight of 0.716 kg. The completed base mix was cooled, packaged in plastic bags, and refrigerated. The mixture of chemopreventive compounds was slugged on a Carver F press. The granules resulting from gently hand grinding in a mortar and pestle were then mechanically blended with core material and excipients, and checked for flow ability in a full size hopper. The granules were then mixed with 250 g of core base mix and excipients then run through the F press again. This required agitation, but the resulting biscuits were consistent in weight ranging from 1.4 to 1.5 g. The samples were checked for compatibility on the Carver press at 10,000 psi with a 1 min dwell time. As a result, the following cancer chemopreventive agents were included in the mixture (mg per one biscuit): resveratrol, 250; ellagic acid, 63; genistein, 125; curcumin, 250; quercetin, 250. Placebo biscuits did not contain the chemopreventive agents.

### Administration of biscuits

In the first phase of the study, canine subjects were given the placebo treat twice per day for a period of three weeks, and blood samples were drawn. Following a two-month resting period, the second phase of the study was launched with the same canine subjects. Over a period of three weeks, the diets of the dogs were supplemented with two biscuit treats containing the chemopreventive agents (Table 1), one biscuit by mouth twice daily approximately 12 hours apart each day. After the administration period, blood was drawn, and the study was terminated.

The owners of these pets transported the dogs to a local veterinary clinic on pre-scheduled days. During these visits, the dogs were weighed and received physical examinations. Venous blood was used for comprehensive serum chemistry profiles and complete blood counts to evaluate organ function and health status, as well as analysis of the chemopreventive agents and assessment of biomarkers. The dogs were maintained by their owners and not housed in any research facility. Special instructions were given as a caution label for biscuits such as keep away from children, store in a refrigerator, may turn urine, feces, saliva yellow, give only as directed, and for animal consumption only.

### Serum and plasma preparation and complete blood counts

Whole blood (6 ml) was collected from each canine subject at the end of the first and second phases of the study. Blood was obtained by venipuncture from either the cephalic or jugular vein (typically with an 18–20 gauge needle attached to a 10 ml syringe). Manual restraint and aseptic techniques were used. The blood from each dog was placed in labelled collection tubes (2 ml in lavender EDTA collection tubes, 1 ml in green lithium heparin collection tubes, and 3 ml in red silicone coated tubes). The lavender tubes were inverted and used to run Complete Blood Counts (CBC) and for the isolation of plasma and lymphocytes. As described below, plasma was derived from the supernatant above the Histopaque-1077 layer used for lymphocyte isolation, and frozen at −80 °C for further analysis. The green tubes were inverted and used to run Comprehensive Chemistry Blood Profiles (CCBP), and the blood within the red tubes was allowed to clot, and then centrifuged for 15 min at 3400 rpm to obtain serum. The serum was frozen prior to analysis by LC-MS/MS. The CBC and CCBPs were run on Abaxis VetScan machines, particularly the VS2 and HM5.

### Lymphocyte isolation and characterization

For the isolation of canine lymphocytes, the procedure of Strasser *et al*.^55^ was followed. Two milliliters of blood in lavender EDTA collection tubes were placed on ice and delivered to the research laboratory. Cold Histopaque-1077 (2 ml; Sigma-Aldrich, St. Louis, MO) solution was carefully place in 15 ml conical centrifugal polypropylene tubes (Becton and Dickinson). Blood was diluted with sterile PBS (1:1) at room temperature, layered carefully onto the Histopaque-1077, and centrifuged at 340 × *g* for 30 min using a swinging-bucket rotor. Centrifugation was terminated without applying a brake. The supernatant (plasma) was frozen at −80 °C, and the opaque bands containing lymphocytes at the interface between plasma and Histopaque-1077 were collected carefully with a siliconized Pasteur pipet and transferred to separate conical centrifugal tubes. Lymphocytes were washed with 10 ml of Hank’s balanced salt solution (HBSS) (two-times; 10 min at room temperature; 300 × g). Cells were counted and frozen as aliquots using preservation media (9 mL FBS mixed with 1 ml sterile dimethyl sulfoxide) at approximately −1 °C/min in an isopropyl freezing container at −80 °C before storage in liquid nitrogen. The average yield was 10^6^ lymphocytes from 1 ml of dog blood.

Cell viability was determined using the Trypan blue exclusion test. Trypan blue stock solution (0.4%; 10 μl) was added to isolated lymphocytes (90 μl) and immediately loaded onto a hemocytometer. The number of blue stained cells and the number of total cells was determined by visual inspection. Viability was found to be 90.2 ± 8.3%.

In addition, lymphocytes were distinguished from monocytes and neutrophils by microscopic inspection after Diff-Quik staining of fixed cell smears. The nucleus of the lymphocyte appears as a dense formation that is oval-round and deep purple. Cytoplasm is clear, with no granules, and only sparse vacuoles. The monocyte nucleus is horse-shoe shaped and not as dense; it is pale purple, lacy and spongy. The cytoplasm appears grey with numerous vacuoles. The nuclei of neutrophils have several lobes, and these cells do not layer above Histopaque-1077 due to their density. Based on this visual inspection, contamination of lymphocytes with other cell types was found to be less than 3%.

### LC-MS and LC-MS/MS

Liquid-liquid extraction was used to prepare dog serum for mass spectrometric analysis. Briefly, 300 μl of serum was mixed with 900 μl of ice-cold acetonitrile. Each sample was vortex mixed for 20 sec and then centrifuged for 10 min at 13,000 × *g*. The supernatant was removed and evaporated to dryness under a stream of nitrogen, and each extract was reconstituted in 100 μl of acetonitrile/water (20:80, v/v) prior to analysis using LC-MS/MS.

Analyses of resveratrol and its metabolites, quercetin and genistein were carried out using a Shimadzu IT-ToF mass spectrometer for purity confirmation and a Shimadzu LCMS-8050 triple quadrupole mass spectrometer for quantitation. Both instruments were equipped with negative ion electrospray and interfaced to Shimadzu HPLC systems (Prominence AR or Nexera, respectively). A Waters (Milford, MA) XBridge C_18_ column (2.1 × 100 mm; 2.5 μm) was used for chromatographic separations. The solvent system consisted of a 5-min linear gradient from 10% to 90% acetonitrile in water at a flow rate of 400 μl/min. The injection volume was 10 μl.

For quantitative analysis, selected reaction monitoring (SRM) was used with collision-induced dissociation and argon as the collision gas. Deprotonated molecules of each analyte were used as precursor ions, and the SRM transitions of the two most abundant product ions were selected as quantifiers and qualifiers, respectively. In some cases, only one SRM transition (quantifier) was used due to the low abundances of other fragment ions. For resveratrol, the SRM transitions were *m/z* 227 to 143 and *m/z* 227 to 185. The SRM transitions of *m/z* 403 to 227 and *m/z* 403 to 143 were used for resveratrol glucuronide, and *m/z* 307 to 227 and *m/z* 307 to 143 were used for resveratrol sulfate. The SRM transitions for genistein were *m/z* 269 to 89 and *m/z* 269 to 187, and the SRM transitions for genistein glucuronide and genistein sulfate were *m/z* 445 to 269 and *m/z* 349 to 269, respectively. Quercetin was measured using the SRM transitions of *m/z* 301 to 151 and *m/z* 301 to 179, and its glucuronide and sulfate metabolites were monitored using the transitions of *m/z* 477 to 301 and *m/z* 381 to 301, respectively. Synthetic isopentyl naringenin was used as an internal standard (SRM transition *m/z* 341 to 119). Although SRM transitions for curcumin (*m/z* 367 to 134 and *m/z* 367 to 217), curcumin glucuronide (*m/z* 543 to 367), curcumin sulfate (*m/z* 447 to 367), and ellagic acid (*m/z* 301 to 229) were monitored, no signals were obtained for these compounds in the study samples.

### Comet assay

**Figure 3:**
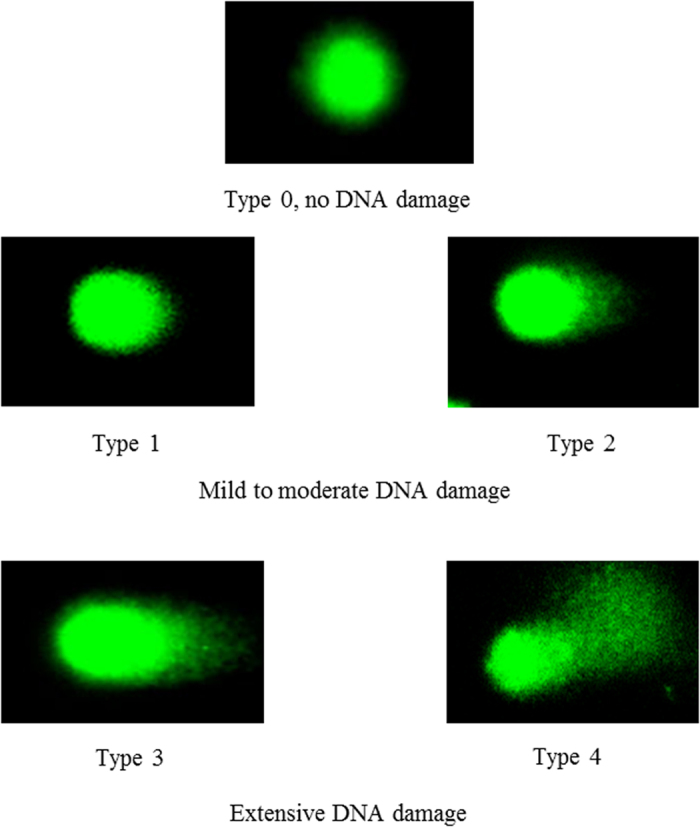
Comet images of lymphocyte DNA damage from control dogs and dogs receiving dietary supplementation. Frozen lymphocytes were thawed, centrifuged, resuspended in PBS and incubated 5 min on ice with 100 μM H_2_O_2_. DNA damage was measured with the comet assay. Type 0, no DNA damage detected in lymphocytes; type 1 and 2 presented at the most in lymphocytes with endogenous DNA damage; type 3 and 4 are in H_2_O_2_ or endonuclease III inducible DNA damage.

The extent of DNA damage in peripheral blood lymphocytes was measured by single-cell gel electrophoresis (the alkaline comet assay)^17^,^56^,^57^. Cells embedded in agarose on a microscope slide are lysed with detergent and high salt to form nucleoids containing supercoiled loops of DNA linked to the nuclear matrix. DNA was allowed to unwind under alkaline conditions. Breaks in the DNA molecule disrupt its complex supercoiling allowing free DNA loops to migrate towards the anode during electrophoresis. DNA damage to the cells can be thus visualized as “comets” (Fig. 3). Two measures of DNA damage in lymphocytes were evaluated: endogenous DNA damage (DNA damage without *ex vivo* H_2_O_2_ exposure) and H_2_O_2_-inducible DNA damage. Analysis was performed according to the method of Singh *et al*.^57^ and Tice *et al*.^58^ with some modifications^59^. A Trevigen Comet assay kit (Gaithersburg, MD) was used for single cell gel electrophoresis. Cells were immobilized in a bed of low melting agarose on a Trevigen CometSlide. A cell suspension containing 1 × 10^5^ cells per ml was combined with 500 μl of LMAgarose providing agarose in a ratio 1:10 for optimal results when spread at 50 μl of mixture per slide well. Slides were covered with a glass cover-slip and left at 4 °C in the dark for 10 min. Increasing gelling time to 30 min improved adherence of the samples. Slides were placed for a minimum of one hour in lysis solution (the buffer formulation is proprietary) to remove membranes, cytoplasm and nuclear proteins. The CometSlide was then immersed in alkaline unwinding solution for 20 min at room temperature or 1 h at 4 °C in the dark. Horizontal electrophoresis was conducted at 21 volts for 30 min at 4 °C. After neutralizing, the gels were soaked in 70% ethanol for 5 min and dried at 37 °C for 10–15 min. Gels were treated with 100 μl SYBR Gold nucleic acid stain (Life Technologies) and viewed by fluorescent microscopy using a Leica TCS SPE confocal microscope (Leica, UK) at Abs/Em 496/540 nm. Slides were examined at 400× magnification using image analysis software (CometScore from TriTek Corp., Sumerduck, VA).

From each replicate slide, 50 nuclei were scored and the percentage of tail DNA intensity was used to evaluate the extent of DNA migration and damage^46^. Fluorescently stained nucleoids from each gel were assessed and classified according to the relative intensity of fluorescence in the tail undamaged (Fig. 3). Each cell was visually scored according to the following criteria: no damage (type 0), mild to moderate damage (type 1 and 2), and extensive DNA damage (type 3 and 4). Under the assay conditions used in this experiment, the intensity of comet tails reflects electrophoretic migration of DNA resulting from strand breaks. This parameter was used as arbitrary units. Tail length tends to increase rapidly with dose at low levels of damage, but soon reaches its maximum. We did not use tail moment units since it combines the information of tail length and tail intensity, but suffers from lack of linearity^46^. To detect specifically oxidized pyrimidines in DNA, recombinant bacterial DNA repair enzyme, endonuclease III from *E. coli*, was used^45^. This provides a specific and sensitive measure of oxidative DNA damage^45^. The slides were washed three-times after lysis for 5 min at 4 °C with buffer (40 mM HEPES-KOH, 0.1 M KCl, 0.5 mM EDTA, 0.2 mg/ml BSA, pH 8). After blotting dry with tissue paper, the gels were incubated for 45 min at 37 °C with either 50 μl buffer or endonuclease III in buffer (1 μg protein/mL). All samples were analysed in duplicate. Levels of endogenous oxidized pyrimidines demonstrated a similar pattern as endogenous DNA damage, i.e., no significant difference between first and second phases (Fig. 1).

### DNA damage inflicted by H_2_O_2_

In the next set of experiments, we employed hydrogen peroxide as an oxidant. It has been shown that hydrogen peroxide causes a dose-dependent increase in DNA strand breaks in human and dog lymphocytes^46^,^60^. Lymphocytes were thawed, mixed with PBS, centrifuged at 200 × g for 3 min at 4 °C, and resuspended in PBS at 2 × 10^6^ cells/ml. For H_2_O_2_ treatment, 10 μl of 1 mM H_2_O_2_ were added to 90 μl of cells in PBS. After 5 minutes on ice, the cells were collected by centrifugation at 300 × g for 10 min and analysed by single cell gel electrophoresis.

### Gene expression analysis

To explore the potential mechanism underlying the protection effect of the dietary supplement on lymphocytes damage caused by H_2_O_2_, a cDNA microarray was employed. For this analysis, RNA was isolated from dog lymphocytes and reverse transcribed to cDNA. The same amount of cDNA with RT^2^ qPCR master mix (25 μl) was placed in each well and subjected to real-time PCR reaction with the SYBR Green detection system. Using the ΔΔCt method we analysed changes in gene expression between the two phases of the study employing Web-based software from SABiosciences (RT^2^ Profiler PCR array data analysis).

The procedure follows. Total RNA was extracted from 1–2 × 10^6^ lymphocytes using Trizol reagent (Invitrogen) according to the method of Chomczynski and Sacchi^61^. Isolated RNA was dissolved in RNase-free water and the quality and quantity were measured using a BioSpec-nano spectrophotometer (ShimadzuBiotech). cDNA was generated from total RNA by reverse transcription using a RT^2^ First strand kit from Qiagen on an ABI 7300 thermocycler (Applied Biosystems Inc.). cDNA was applied to a RT^2^ profiler custom made PCR array related to dog oxidative stress (Table 5) following the manufacturer’s instructions (QIAGEN-Frederick, SABiosciences). Samples were run in triplicate to ensure amplification integrity. Expression levels were analysed with each sample^62^,^63^. Fold-changes of genes were determined and visualized as heatmaps (red: up-regulation, and green: down-regulation) (Fig. 2).

### Statistical analysis

Results are presented as means ± SE. Data representing the various groups were compared using the Student’s *t*-test and the level of P < 0.05 was considered as significantly different. Other statistical considerations are described in the text or tables.

## Additional Information

**How to cite this article**: Kondratyuk, T. P. *et al*. Evidence supporting the conceptual framework of cancer chemoprevention in canines. *Sci. Rep.* **6**, 26500; doi: 10.1038/srep26500 (2016).

## Figures and Tables

**Table 1:** Chemopreventive compounds administered to dogs.

Compound	Source	Purity	Dose (g/dog/day)	Dose in human studies (g/person/day)
Resveratrol	Hangzhon Dayangchem Co., Shanghai, China	>95%	0.5	1.0
Ellagic acid	Shandong Juye Sunnyfarm Natural Product CO., LTD, Shandong, China	>99%	0.125	0.25
Genistein	Shandong Juye Sunnyfarm Natural Product CO., LTD, Shandong, China	>95%	0.250	0.5
Curcumin	Shandong Juye Sunnyfarm Natural Product CO., LTD, Shandong, China	>95%	0.5	1.0
Quercetin	Hangzhon Dayangchem Co., Shanghai, China	>95%	0.5	1.0

**Table 2:** Comprehensive blood profiles.

	Reference range	First phase	Second phase
ALB	2.5–4.4 g/dL	3.6 ± 0.1	3.6 ± 0.1
ALP	20–150 U/L	45.1 ± 6.9	45.5 ± 6.1
ALT	10–118 U/L	53.1 ± 9.4	69.1 ± 11.5
AMY	200–1200 U/L	511.1 ± 53.6	538.6 ± 58.7
TBIL	0.1–0.6 mg/dL	0.3 ± 1.0	0.3 ± 0
BUN	7–25 mg/dL	14.3 ± 1.0	15.4 ± 1.2
CA^++^	8.6–11.8 mg/dL	10.6 ± 0.1	10.6 ± 0.1
PHOS	2.9–6.6 mg/dL	3.7 ± 0.2*	4.6 ± 0.1*
CRE	0.3–1.4 mg/dL	1.1 ± 0.1	1.0 ± 0.1
GLU	60–110 mg/dL	104.6 ± 4.0	104.4 ± 4.5
NA^+^	138–160 mmol/L	144.0 ± 0.7	145.3 ± 0.3
K^+^	3.7–5.8 mmol/L	4.7 ± 0.2	4.2 ± 0.1
TP	5.4–8.2 g/dL	6.7 ± 0.1	6.7 ± 0.1
GLOB	2.3–5.2 g/dL	3.1 ± 0.1	3.3 ± 0.1

ALB - albumin; ALP -alkaline phosphatase; ALT - alanine aminotransferase; AMY - amylase; TBIL - total bilirubin; BUN - blood urea nitrogen; CA^++^ - calcium; PHOS - phosphorus; CRE - creatinine; GLU - glucose; NA^+^ - sodium; K^+^ - potassium; TP - total protein; GLOB – globulin. *p < 0.01.

**Table 3:** Complete blood count.

	Reference range	First phase	Second phase
GRA	3.0–12.0 × 10^9^/L	8.4 ± 0.71	9.9 ± 0.8
WBC	6.0–17.0 × 10^9^/L	11.1 ± 1.1	12.7 ± 1.2
LYM	1.0–4.8 × 10^9^/L	2.2 ± 0.6	1.9 ± 0.5
MON	0.2–1.5 × 10^9^/L	0.5 ± 0.1*	0.9 ± 0.1*
RBC	5.5–8.5 × 10^12^/L	7.8 ± 0.3	7.4 ± 0.3
HGB	12.0–18.0 g/dL	16.9 ± 0.6	17.0 ± 0.4
HCT	37.0–55.0%	49.2 ± 1.9	49.3 ± 2.1
MCV	60–77 fl	65.6 ± 1.2	66.3 ± 1.1
MCH	19.5–24.5 pg	23.2 ± 1.0	23.0 ± 0.4
MCHC	31.0–34.0 g/dL	34.3 ± 0.5	34.7 ± 0.7
PLT	200–500 × 10^9^/L	233.8 ± 25.5	272.6 ± 59.4
MPV	3.9–11.1 fl	10.1 ± 0.4	9.9 ± 0.4

GRA - granulocytes; WBC - white blood cells; LYM - lymphocytes; MON - monocytes; RBC - red blood cells; HGB - hemoglobin; HCT - hematocrit; MCV - mean corpuscular volume; MCH - mean corpuscular hemoglobin; MCHC - mean corpuscular hemoglobin concentration; PLT - platelets; MPV - mean platelet volume; *p < 0.0001.

**Table 4:** Concentrations of chemopreventive compounds and its metabolites in dog serum.

Compounds	Concentration in serum, ng/ml First phase	Concentration in serum, ng/ml Second phase
Resveratrol	N/D	19.3 ± 11.02^a^
Resveratrol-3-*O*-glucuronide	N/D	64.29 ± 27.24^a^
*trans-*Resveratrol-4′-sulfate	N/D	44.43 ± 12.23^a^
*trans-*Resveratrol-3-sulfate	N/D	424.38 ± 80.40^a^
*cis-*Resveratrol-3-sulfate	N/D	13.63 ± 5.27^a^
Resveratrol sulfate total	N/D	482.43 ± 75.60^a^
Ellagic acid	N/D	N/D
Genistein	N/D	N/D
Genistein-4′-glucuronide	327.72 ± 211.13^a^	7083.7 ± 128.18*
Genistein-7-glucuronide	N/D	861.74 ± 329.6
Genistein sulfate	961 ± 680.3	26958.6 ± 2300**
Curcumin	N/D	N/D
Curcumin glucuronide	N/D	N/D^b^
Quercetin	N/D^c^	2 ± 1

N/D – none detected.

^a^One dog did not show any resveratrol, genistein, or respective metabolites, in blood serum, and was excluded from the analysis.

^b^Two dogs had concentrations of curcumin glucuronide of 94 and 31 ng/ml.

^c^Three dogs had concentrations of quercetin of 2, 3 and 2 ng/ml, all others dogs had below detectable level; *p = 0.0018; **p = 0.003.

**Table 5:** Oxidative stress related genes custom made for dogs.

GeneBank	Symbol	Description
NM_001003026	ALB	Albumin
XM_536613	ALOX12	Arachidonate 12-lipoxygenase
XM_845955	AOX1	Aldehyde oxidase 1 pseudogene
XM_860950	APOE	Apolipoprotein E
NM_001003119	ATOX1	ATX1 antioxidant protein 1 homolog (yeast)
XM_845605	BNIP3L	BCL2/adenovirus E1B 19 kDa protein 3-like
NM_001002984	CAT	Catalase
NM_001003010	CCL5	Chemokine (C-C motif) ligand 5
NM_001194970	CCS	Copper chaperone for superoxide dismutase
NM_001100290	CYBA	Cytochrome b-245, alpha polypeptide
NM_001100291	CYBB	Cytochrome b-245, beta polypeptide
NM_001077587	CYGB	Cytoglobin
XM_546693	DHCR24	24-dehydrocholesterol reductase
NM_001003122	DUOX1	Dual oxidase 1
XM_005638367	DUOX2	Dual oxidase 2
XM_005619446	DUSP1	Dual specificity phosphatase 1
XM_005635675	EPHX2	Epoxide hydrolase 2, cytoplasmic
XM_548229	EPX	Eosinophil peroxidase
XM_005637294	FOXM1	Forkhead box M1
NM_001003080	FTH1	Ferritin, heavy polypeptide 1
XM_003431750	GCLC	Glutamate-cysteine ligase, catalytic subunit
XM_005621897	GCLM	Glutamate-cysteine ligase, modifier subunit
NM_001115119	GPX1	Glutathione peroxidase 1
NM_001115135	GPX2	Glutathione peroxidase 2 (gastrointestinal)
NM_001164454	GPX3	Glutathione peroxidase 3
NM_001003213	GPX5	Glutathione peroxidase 5 (epididymal androgen- protein)
NM_001256320	GPX6	Glutathione peroxidase 6 (olfactory)
XM_005629410	GPX7	Glutathione peroxidase 7
NM_001252323	GPX8	Glutathione peroxidase 8 (putative)
XM_003432097	GSR	Glutathione reductase
XM_005634964	GSS	Glutathione synthetase
NM_001252167	GSTP1	Glutathione S-transferase pi 1
XM_003435052	GSTZ1	Glutathione transferase zeta 1
XM_005620945	GTF2I	General transcription factor IIi
NM_001194969	HMOX1	Heme oxygenase (decycling) 1
NM_001003392	KRT1	Keratin 1
XM_005627164	LOC102155697	Heat shock 70 kDa protein 1-like
XM_844054	LOC607408	BCL2/adenovirus E1B 19 kDa interacting protein 3
XM_003433333	LOC608103	Methionine sulfoxide reductase A
XM_548231	LPO	Lactoperoxidase
XM_005625921	MB	Myoglobin
NM_001252410	MGST3	Microsomal glutathione S-transferase 3
XM_847352	MPO	Myeloperoxidase
XM_005630257	MPV17	MpV17 mitochondrial inner membrane protein
XM_844481	NCF1	Neutrophil cytosolic factor 1
NM_001101832	NCF2	Neutrophil cytosolic factor 2
NM_001003186	NOS2	Nitric oxide synthase 2, inducible
XM_005633778	NOX4	NADPH oxidase 4
NM_001103218	NOX5	NADPH oxidase, EF-hand Ca binding domain 5
XM_848524	NQO1	NAD(P)H dehydrogenase, quinone 1
XM_547012	NUDT1	Nudix (nucleoside diphosphate moiety X)-type motif 1
XM_003431771	OXR1	Oxidation resistance 1
XM_005634251	OXSR1	Oxidative-stress responsive 1
XM_534974	PDLIM1	PDZ and LIM domain 1
XM_005616288	PNKP	Polynucleotide kinase 3′-phosphatase
NM_001252165	PRDX1	Peroxiredoxin 1
XM_542042	PRDX2	Peroxiredoxin 2
NM_001256485	PRDX3	Peroxiredoxin 3
XM_548896	PRDX4	Peroxiredoxin 4
XM_005631542	PRDX5	Peroxiredoxin 5
XM_537190	PRDX6	Peroxiredoxin 6
XM_543041	PREX1	Phosphatidylinositol-3,4,5-dependent Rac exchange factor 1
NM_001013423	PRNP	Prion protein
NM_001003023	PTGS1	Prostaglandin-endoperoxide synthase 1
NM_001003354	PTGS2	Prostaglandin-endoperoxide synthase 2
XM_005637964	PXDNL	Peroxidasin homolog (Drosophila)-like
XM_543225	SCARA3	Scavenger receptor class A, member 3
NM_001115118	SEPP1	Selenoprotein P, plasma, 1
XM_546184	SFTPD	Surfactant protein D
XM_850289	SIRT2	Sirtuin 2
NM_001003035	SOD1	Superoxide dismutase 1, soluble
XM_533463	SOD2	Superoxide dismutase 2, mitochondrial
XM_545973	SOD3	Superoxide dismutase 3, extracellular
XM_005626348	SQSTM1	Sequestosome 1
XM_005635344	SRXN1	Sulfiredoxin 1
NM_001286859	STK25	Serine/threonine-protein kinase 25
NM_001003009	TPO	Thyroid peroxidase
XM_535981	TTN	Titin
XM_533037	TXNIP	Thioredoxin interacting protein
NM_001122673	TXNRD1	Thioredoxin reductase 1
XM_845088	TXNRD2	Thioredoxin reductase 2
NM_001122778	TXNRD3	Thioredoxin reductase 3
NM_001003048	UCP2	Uncoupling protein 2 (mitochondrial, proton carrier)
NM_001114757	VIMP	Selenoprotein S

**Table 6:** Transcriptional regulation of oxidative stress-related genes in dog lymphocytes.

Gene	Description	Function	Fold Induction	*P*value
	Upregulated genes			
*CYBB*	Cytochrome b-245, beta polypeptide (chronic granulomatous disease)	Antioxidant	2.26	0.051
*DUSP1*	Dual specificity phosphatase 1	Oxidative stress responsive gene	2.68	0.023
*GSR*	Glutatione reductase	Oxidative stress responsive gene	2.34	0.016
*UCP2*	Uncoupling protein 2(mitochondrial, proton cattier)	Superoxide metabolism	3.54	0.044
*VIMP*	Selenoprotein S	Oxidative stress responsive gene	3.62	0.022
	Downregulated genes			
*ATOX1*	ATX1 antioxidant protein	ROS metabolism	−2.83	0.048
*CCL5*	Chemokine (C-C motif) ligand 5	Oxidative stress responsive gene	−2.28	0.018
*EPX*	Eosinophil peroxidase	Antioxidant	−2.43	0.046
*MPV17*	MpV17 mitochondrial inner membrane protein	ROS metabolism	−3.51	0.04
*PRNP*	Prion protein	Oxidative stress responsive gene	−2.85	0.047
*SOD3*	Superoxide dismutase 3, extracellular	Antioxidant	−3.28	0.035

Positive values indicate up-regulation; negative values, down-regulation.
